# Measuring Research Information Citizenship Across ORCID Practice

**DOI:** 10.3389/frma.2022.779097

**Published:** 2022-03-28

**Authors:** Simon J. Porter

**Affiliations:** Digital Science, London, United Kingdom

**Keywords:** ORCID, scientometrics, Dimensions, research infrastructure, scholarly communications

## Abstract

Over the past 10 years, stakeholders across the scholarly communications community have invested significantly not only to increase the adoption of ORCID adoption by researchers, but also to build the broader infrastructures that are needed both to support ORCID and to benefit from it. These parallel efforts have fostered the emergence of a “research information citizenry” between researchers, publishers, funders, and institutions. This paper takes a scientometric approach to investigating how effectively ORCID roles and responsibilities within this citizenry have been adopted. Focusing specifically on researchers, publishers, and funders, ORCID behaviors are measured against the approximated research world represented by the Dimensions dataset.

## 1. Introduction

In 2012, the founding members of ORCID Consortium asked the scholarly community to join them in imagining a new version of the scholarly record: One in which researchers were globally and uniquely identified (Haak et al., 2012). Although this sounds like a simple, incremental step, it was much more fundamental, at once solving information ambiguities and addressing issues of identity in an increasingly international community where trust in the validity of authorship is a critical currency. On a practical level, by attaching their ORCID iD to research objects such as publications, researchers would be able to reduce the administrative burden of communicating who they are and what they do across multiple domains including publishing, institutional assessment, research funding, and scholarly information discovery. Institutions within these domains would, in turn, gain greater strategic insight from the scholarly record not readily realizable within their own information silos.

Even at the beginning of the ORCID project, it was understood that to realize the benefits of ORCID, social and cultural change would be required in addition to technical change. Sustained community investment and collaboration around the development of ORCID and related infrastructures would need to be established amongst a disparate group of stakeholders with different drivers and motivations. All would need to be committed to developing and adopting new workflows and methods of information exchange. By connecting themselves to, and relying on each other, this newly networked community of researchers, institutions, funding bodies, publishers, and research service providers would establish the foundations of a new research information citizenship (Porter, 2016), defined by researcher agency, and distributed metadata stewardship.

When we speak about researcher agency we are specifically referring to the combination of a researcher-owned digital representation in the form of an ORCID record together with the set of interactions with the digital world through that representation. By implicitly establishing this as the de facto definition of researcher agency, ORCID upended passive assumptions about how a research identifier could be deployed. An ORCID iD was not just an identifier for a researcher that could be added by anybody to a record, it simultaneously served as an identity through which a researcher could exert digital agency—this constituted a major step in establishing an infrastructural norm in the emergent digital research landscape. In addition to creating trusted assertions within publisher, funder, and other administrative workflows, a researcher could also gain access to research services. These services could include research facilities and collaboration tools, both at an administrative level of securing that access, as well as at the practical level of logging into a piece of equipment to perform their work. This merging of the worlds of describing research and conducting it created the possibility that trusted metadata about who was doing what research could be a byproduct of research itself.

Distributed metadata stewardship arises as a natural consequence of researcher agency in a complex ecosystem of stakeholders: It is simply not efficient, desirable, or practical to try to centralize permissions and the transaction logs associated with intrinsically distributed activities (typically those where researchers usually transact with any number of distributed stakeholders)^1^. As researchers engage across the activities in the research life cycle, different parts of the data contained in the ORCID registry of scholarly activities are made available to, and shared across, many different systems. In the case of publishing, a set of authenticated ORCID relationships between a set of researchers and a publication is collected at the time of submission or during the publication process. This distributed authentication is important as capturing these relationships at the point of submission is one of the few times when there is an incentive that can be applied in favor of data quality. A further consequence of distributed metadata stewardship is that the scholarly record itself becomes distributed, with different stakeholders holding differing levels of detail about each ORCID in their own systems. For instance, a publication identified by a DOI supplied by Crossref records the link between an ORCID iD and a specific author on the paper (Clark, 2020), whilst an ORCID record at orcid.org records the direct connection between a publication and researcher (ORCID, 2021b). While the distributed nature of this approach to data holding adds a level of privacy for an individual (since no one actor or system has access to all the information about that individual) there are also pitfalls - specifically, the opportunity for data loss or data inconsistency. Without a single source of truth or a set of mechanisms to homogenize data (such as a distributed data ledger), there is always the possibility of data ambiguity.

In addition to changes in workflows and responsibilities, global adoption of ORCID has also required a global network of change agents. Rather than being “top-down” initiatives led by governments, the mainstay of these activities has been done slowly with a mixture of bottom-up approaches and mid-level interventions. Country-led ORCID Consortia have organized to help researchers understand the benefits of maintaining their ORCID record. For their part, funders and publishers initially made ORCID optional in their grant and publication submission processes. In the last few years this has increasingly moved to requiring researchers to supply their ORCID as part of these processes (ORCID, 2016). Some countries have also chosen to act at a higher level and now mandate the use of ORCID iDs as part of their researcher reporting processes (Puuska, 2020).

While nudges and mandates can be powerful in gaining adoption, it is easier to achieve compliance if there is a tangible benefit to researchers and other stakeholders. In parallel with the development of the technology and compliance landscape, infrastructure has been developed to facilitate these benefits. Change has not been uniform, with funders and publishers moving toward ORCID support at different rates depending on their capacity to change their systems to conform with ORCID best practice (Mejias, 2020).

Almost a decade on and the success of ORCID can readily be measured by the number of participants actively engaged with ORCID. In 2018, UNESCO reports that the global researcher population had reached 8.9 Million FTE (UNESCO, 2021). At the end of 2018, there were 5.8 Million live ORCID registrations, 1.4M of whom had recorded at least one work (ORCID, 2021a). By July 2021, the number of ORCIDs that had an authenticated relationship with at least one scholarly work had increased to 3.9M. That these numbers are even within the same order of magnitude as the UNESCO figure is a significant achievement. While compelling, what these headline numbers do not indicate is the degree to which behavior and citizenship around ORCID research information has changed. Gaining an insight into the following questions would provide a better understanding of how far research citizenship now extends: Are researchers actively using their ORCID throughout the research process, or does the observed behavior simply reflect a compliance response to mandates? Beyond the ORCID registry itself, how are the responsibilities of distributed metadata stewardship being met? Does behavior differ between countries and disciplines? How far have publishers changed their practices to accommodate ORCID workflows? What is the quality of ORCID metadata outside of the ORCID registry (particularly in the Crossref registry)?

To address these questions, this paper takes a scientometric (Leydesdorff, 1995) approach and analyses ORCID behaviors with reference to the approximated world of researchers as embodied in the Dimensions database. Although not 100% accurate for all the reasons that ORCID was created in the first place, Dimensions provides a global set of algorithmically created researcher identities against which ORCID uptake can be measured. Additionally, Dimensions global coverage of publications and grants and the links between them provides a sufficient background dataset against which to conduct the analysis. Section 2 of this paper provides a description of the methodology used to link ORCID assertions from both Crossref and ORCID with the Dimensions dataset. Section 3 provides an analysis of the ORCID behaviors that we are able to observe. Finally, Section 4 reflects on the consequences of these findings.

## 2. Methods

A previous analyses of ORCID uptake and usage used ORCID's public data file and publication level integration with metadata from Web of Science (Dasler et al., 2017). Comparative observations about researcher population by discipline and country were made by using reference researcher populations that were created programmatically from the Web of Science dataset by the Centre for Science & Technology Studies (CWTS) at Leiden University. In this investigation we have used the combined ORCID statements from both the ORCID (Blackburn et al., 2020) and Crossref public files (Clark, 2021) to examine ORCID-related behavior in publishing as a whole. This distinction is significant as it allows the flow of ORCID records between Crossref and the ORCID registry to be observed. Our approach also differs from the previous analysis in that we have integrated researcher identities from Dimensions, as well as matching records at the publication level. Integrating ORCID and Dimensions researcher identities allows for measures of individual record completeness to be approximated. Since the original study several large-scale initiatives have had an impact on ORCID adoption including funder and publisher mandates. Dimensions is well suited to provide insights into these developments as both funders and publishers are uniquely identified, allowing for publications to be easily aggregated and analyzed along these axis. The methodology for integrating the three datasets is described below.

### 2.1. Data Integration

To begin our analysis we needed to create a baseline dataset to facilitate comparisons. We generated this baseline by integrating Crossref and ORCID data with Dimensions (Hook et al., 2018) so that researchers without ORCID iDs could be identified. Inclusion of the Dimensions data allows us to access enhanced metadata concerning author affiliations, as well as publisher-level and funder-level information. Dimensions serves as a convenient intersection between the Crossref and ORCID datasets since the construction of Dimensions is predicated on persistent unique identifiers (PIDs) with information from orcid.org already matched back to Dimensions, and the Crossref data forming a key part of Dimensions' publications data spine (Visser et al., 2021). Data from the Crossref public file can be easily integrated at the author level, as the author level names largely match those in Dimensions. ORCID and Crossref data were loaded into Google BigQuery, allowing easy integration with Dimensions data, which is also available as a Google BigQuery dataset (Hook and Porter, 2021).

Table 1 provides a breakdown of the fields used in the analysis. Data was analyzed along the following axis: Publication, Researcher Affiliation (Country), Publisher, Funder, and Researcher Discipline. Of these, Publisher, Funder, and Researcher Discipline are described in further detail below.

**Table 1:** Data sources and fields used in the analysis.

**Source**	**Entity**	**Metadata analyzed**
ORCID	Researcher	First name, last name, ORCID, date ORCID created
ORCID	Publication	DOI
Crossref	Publication	DOI
Crossref	Author.	First name, last name, ORICD iD
Dimensions	Researcher	Researcher_id, ORCID iD, and most recent institution & country affiliation
Dimensions	Author	First name, last name
Dimensions	Publisher	Publisher and journal references
Dimensions	Funder	Links between funders and researcher

### 2.2. Publications

Publication data from Crossref was integrated with publication data in Dimensions by matching on DOI, first name and surname. Reflecting the differences in metadata schemas, publications in the ORCID registry were not matched at the author level, but instead on ORCID iD and DOI. Publications without Crossref DOIs were also ignored as they did not have bearing on the practices measured in this investigation.

### 2.3. Researchers

Having matched Publications from Dimensions and Crossref at the author level, the corresponding researcher_id (Dimensions), and ORCID iD (Crossref) could be associated. This match could only be done after having addressed a data quality issue in the Crossref file (described below).

### 2.4. Affilitions

For this analysis the richer set of information around affiliation data in the ORCID record was not used in favor of Dimensions data that provided a consistent method of assigning institutional affiliation across researchers with and without ORCID iDs. The most recent affiliation for a researcher was calculated based on the affiliations associated with their most recent publications and grants.

### 2.5. Researcher Discipline

To facilitate the analysis of ORCID adoption by discipline, a researcher's discipline was defined as the two-digit Field of Research classification (Australian Bureau of Statistics, 2020) in which they most commonly publish (Porter, 2021). These classifications were assigned to publications using an NLP approach, ensuring consistency across a global dataset.

### 2.6. Data Quality

**Figure 1:**
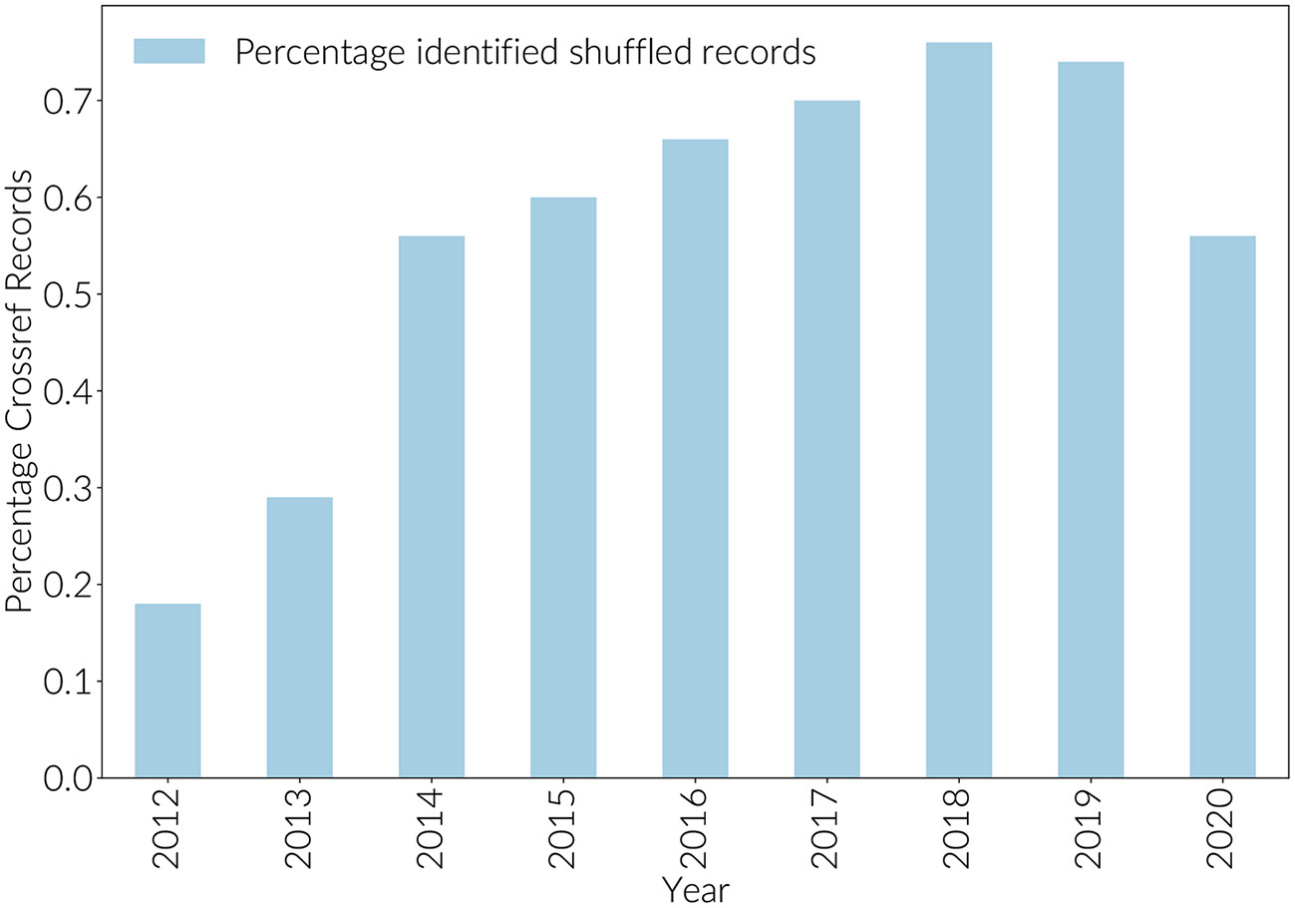
Percentage identified shuffled ORCID assertions.

Before integrating Crossref and ORCID author assertions with Dimensions, Crossref records were first adjusted to address the phenomenon of “author shuffling.” (Author shuffling is an effect where by an ORCID iD is assigned to the wrong author on a paper Baglioni et al., 2021). By joining raw Crossref records to Dimensions records, it was possible to estimate the size of the author shuffling problem by identifying papers where authors appeared to be collaborating with themselves. In the case of author shuffling, for an author with a reasonably sized publication history, an ORCID iD will be matched to more than one Dimensions researcher_id. For shuffled records, the research_id to which they are matched will be one of their collaborators. Shuffled records can be identified when more than one of the researcher_ids that the ORCID has been associated with appears on the same paper. As Figure 1 shows, the percentage of shuffled records in Crossref rose to just over .7% in 2018 before dropping slightly to approximately 0.5% in 2020. This is almost certainly an underestimate as this method only identifies cases where Dimensions has a researcher_id for the shuffled author as well as the actual author.

To increase the chances of finding all shuffled records so that they could be cleaned before matching, suspect author assertions were identified based on the following criteria:

1. The author appears to be collaborating with themselves (as above), or the match with Crossref results in more than one ORCID iD being assigned to a researcher_id;
2. The ORCID iD author matched identified by Dimensions disagrees with the author ORCID assertion in Crossref;
3. Dimensions does not have a researcher_id for the author ORCID assertion in Crossref.

For these records, a simple string matching algorithm using a Levenshtein Distance calculation was used to establish the most likely match between the name recorded in the ORCID record, and the names of the author on the paper (Cohen, 2015). If this approach returned the same match as Crossref with a ratio score of greater than or equal to 70%, the Crossref match was kept. If the name could be matched to another author on the paper with a confidence score of greater than 90%, then the ORCID author assertion was reassigned to that author. The difference in confidence cutoffs places a value on the Crossref assertion, as well as addresses a problem with the matching approach that gave very high scores to incorrectly matched authors with very short first names and surnames.

One drawback of the above approach to fixing shuffled records is that it creates a bias against some of the very use cases that ORCID was established to help solve, including changes in married names, names with few characters, and names with non-Latin characters. In addition, some authors used the native version of their name in their ORCID record, but published with the anglicized version. To help reduce the number of times these instances were rejected due to low name matching scores, author name ORCID matches that could be found across publications from multiple publishers were also accepted as true.

Using the combination of these methods, 1.7% connections asserted in the Crossref data were removed, and 0.5% reassigned to other authors. That 1.2% of connections were not easily recoverable is illustrative of the difficulty of name matching based on strings.

## 3. Results

### 3.1. ORCID Adoption and Engagement

With the integrated ORCID, Crossref, and Dimensions datasources, we are able to measure ORCID adoption as the percentage of researchers in a given year who have at least one publication with a DOI linked to their ORCID iD either in ORCID directly or identified within the Crossref file. ORCID record completeness was also approximated by comparing the number of publications linked to an ORCID iD vs. the number of publications linked to the Dimensions researcher_id against which the ORCID identifier was matched. As defined, ORCID *adoption* is intended as a measure of active usage, whereas ORCID record *completeness* is a proxy for engagement.

Researchers with only a few publications are difficult to identify algorithmically as there are few data points to base a decision on. To increase the chances of Dimensions accurately identifying researchers, researchers with less than 5 years publishing history have been excluded from the analysis. Completeness calculations have also been restricted to publications between 2015 and 2019.

We argue that completeness can be thought of as a proxy for engagement, since a researcher needs to take responsibility for their own record in order for it to be maintained accurately. Firstly, they must set up their ORCID to receive automatic updates from Crossref, and secondly, they must update their own record with ORCID publication assertions not captured during publisher submission. By including publications in the Crossref record, this measure of completeness is able to include ORCID assertions are not present in a researcher's public record. ORCID assertions that have been made private by the researcher and are not included in the Crossref record have not been included in the analysis.

#### 3.1.1. ORCID Adoption and Engagement by Country

**Figure 2:**
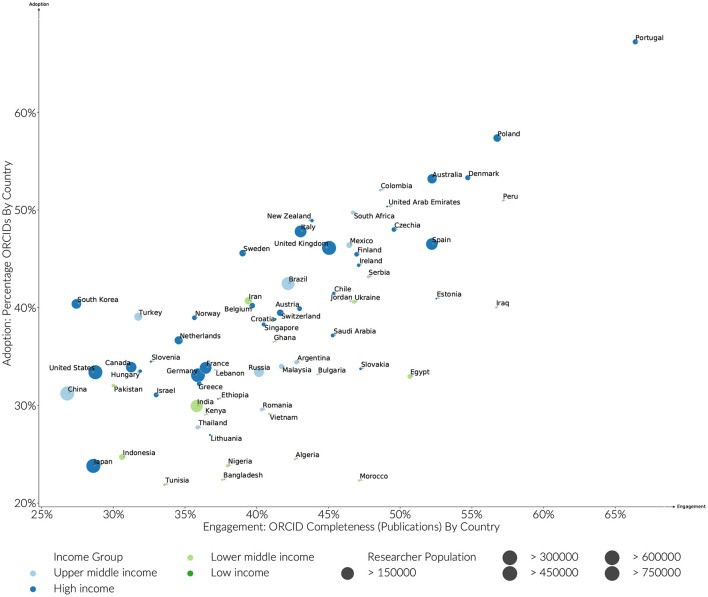
Estimated ORCID adoption and engagement by country. Active researchers in the analysis must have (A) published between 2015 and 2019, (B) have a publication history of greater than 5 years, and (C) published more than 5 papers.

Breaking measures of ORCID adoption and completeness down, by Country (Figure 2), it is clear that just as factors other than economic wealth strongly influence the scientific wealth of nations (Allik et al., 2020), local research environments significantly influence ORCID researcher engagement. Looking at the years between 2015 and 2019, Portugal ranks most highly in both Adoption (67%), and Engagement (70%). Poland, Australia, Denmark, Columbia and South Africa and New Zealand then follow with adoption levels between 50 and 60%. Of the countries with an identified researcher pool of > 100,000, the more established and larger scale research economies, Italy, Spain, and the United Kingdom have adoption rates in the region just below or just above 40%. However, not all the established research economies show the same level of engagement for a cadre of different reasons: The United States, China, and Japan are notable for their relatively low adoption and engagement rates compared to countries in the same World Bank income bands. In the case of the United States, this is likely to be due to the lack of centralized, government-led research evaluation and levers associated with block funding the other countries such as those mentioned have available. Japan has adopted its own system of researcher identification with the researchmap.jp system, which stands apart from all other global systems. China, while moving quickly, is simply at an earlier stage of engagement with globalized research infrastructure and has unique challenges in terms of name disambiguation.

**Figure 3:**
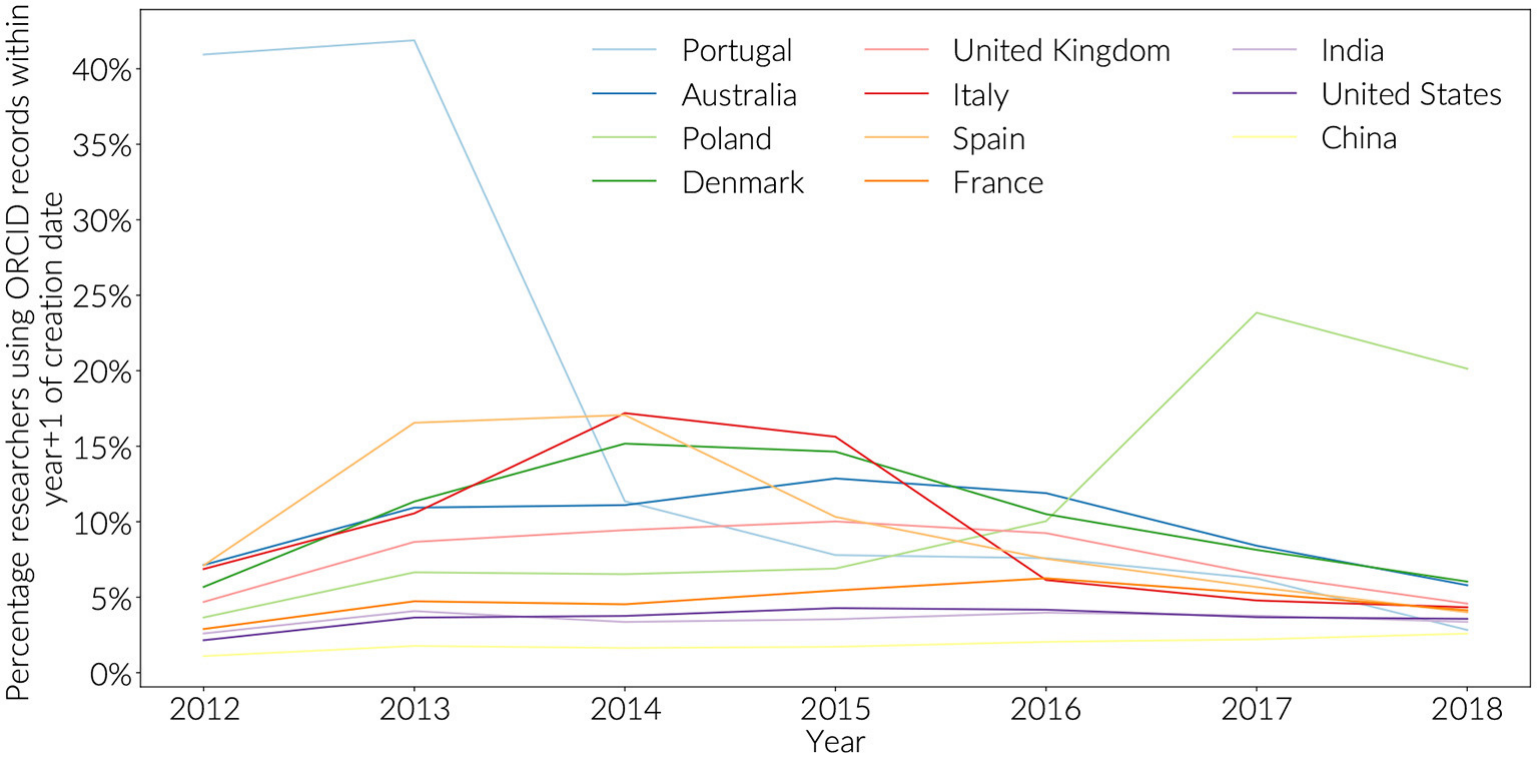
New ORCID registrations by year (+1). Totals are not cumulative, showing early peaks in adoption.

Countries with high engagement have also demonstrated concerted enrolment efforts. These efforts can be detected in the publication record by looking for ORCID iDs that are used in publications between the time they were created and the end of the next full publication year (Figure 3). Using this methodology, it is possible to observe that Portugal started early with a concentrated effort in 2012, and 2013 at the launch of the ORCID initiative, with Spain following over 2013 and 2014, Italy and Denmark in 2014-2015. Both Australia and the United Kingdom showed a sustained engagement at or slightly below 10% between 2013 and 2016. Poland is distinct in initiating renewed engagement activities in 2016.

**Figure 4:**
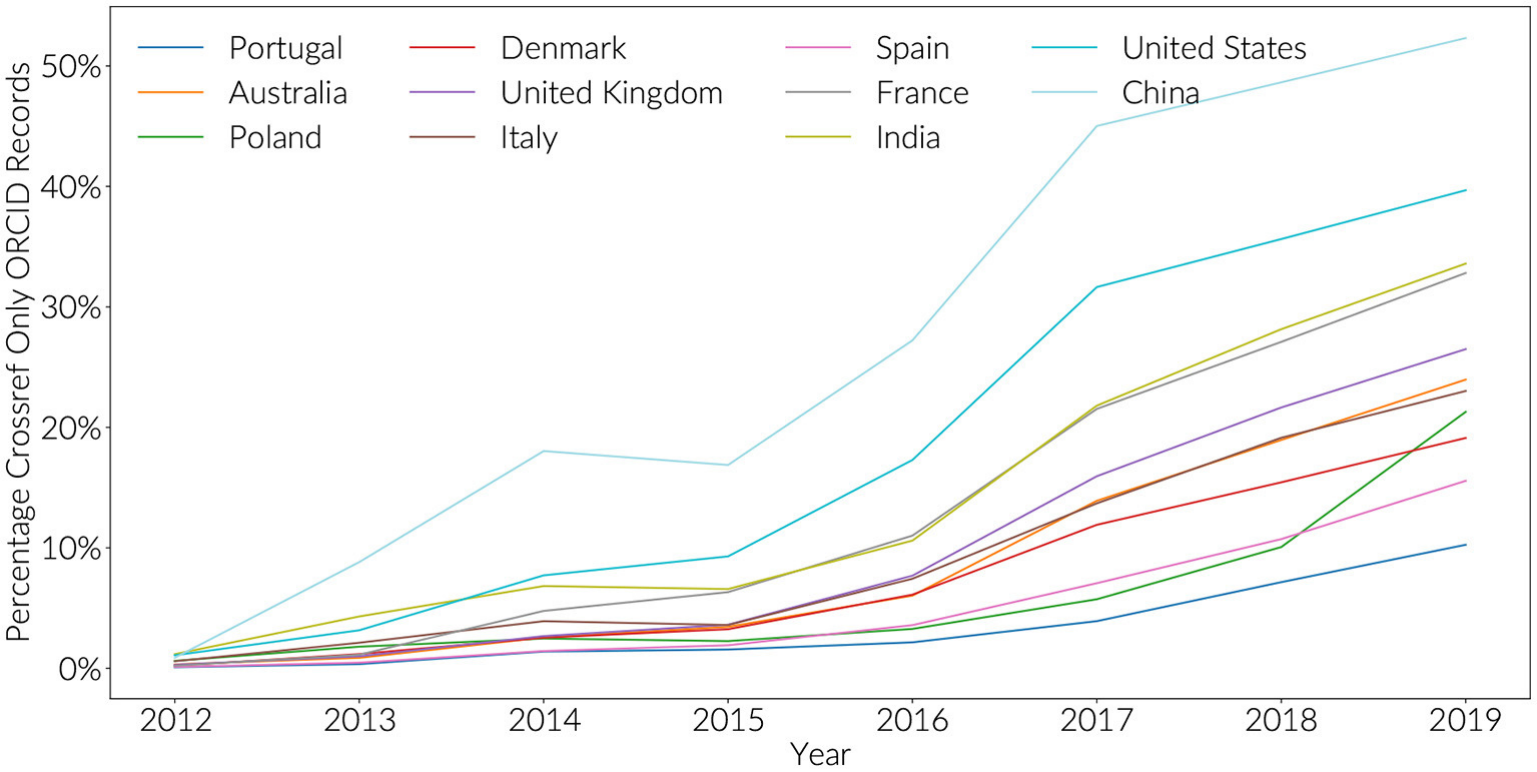
Percentage of ORCID records with only Crossref assertions by year.

Countries with low engagement show a different pattern (Figure 4). Since 2016, there has been a steep increase in ORCID iD assertions that are present in Crossref, but are not displayed in a researcher's own ORCID record. This is particularly prominent with Chinese authors where 50% of researchers in 2019 do not have any 2019 statements from Crossref that have made it back to their public ORICD record. For United States authors, this value 40%, compared to 10% for Portugal, and just above 20% for Italy and Australia. This result is despite the fact that there is an established workflow to push ORCID assertions back from Crossref to ORCID, and that all researchers are required to do is to provide consent in response to an email (Brown et al., 2016). At least two scenarios might explain this behavior with the strength of this effect varying by country:

1. An increasing number of researchers are registering for an ORCID iD because they are encouraged to during early career studies or because they need one to engage in certain formal processes within their country. Motivated from a position of compliance, these researchers are not sufficiently engaged to go further and keep their ORCID record up to date either by entering in details directly, or by authorizing the systems that they engage with to update their record on their behalf (such as the Crossref auto update functionality.) (Mejias, 2020).
2. An increasing number of researchers are choosing to keep their record private due to growing privacy concerns associated with digital existence as a whole.

The first scenario is concerning. It suggests that a growing number of researchers will not be able to use their ORCID iD as a tool to reduce academic burden. These researchers will likely be frustrated when the act of supplying their ORCID iD in a funder workflow does not result in their record being populated. This scenario is reasonably likely. In 2017, after the initial release of the Crossref auto-update functionality, only 50% of researchers were reported as choosing to respond to the email from Crossref offering to auto update their ORCID record when new publications were detected (Meadows and Haak, 2017). For some countries, it does not appear as if this number has significantly improved since this time.

The second scenario, although not necessarily preventing any ORCID use cases, would indicate an increasing desire by researchers not to be ‘known' by their ORCID iD, and perhaps a lack of buy-in to open identifier infrastructure. Both scenarios would be regional examples of less than enthusiastic research information citizens.

Part of difference between country cultures can be explained by the interventions local funding agencies have made in integrating ORCID iDs into their processes. Funding agencies can impact ORCID behavior by requiring researchers to have an ORCID (adoption,) as well as by driving engagement by making it easy for researchers to use information from their ORCID records in their publications, or implying a strong preference for complete ORCID records. Beyond publication workflows, funders will also play an increasing role in linking ORICD iDs to open public records of grants (ORCID Funder Working Group, 2019) creating similar data reuse patterns to publications.

**Figure 5:**
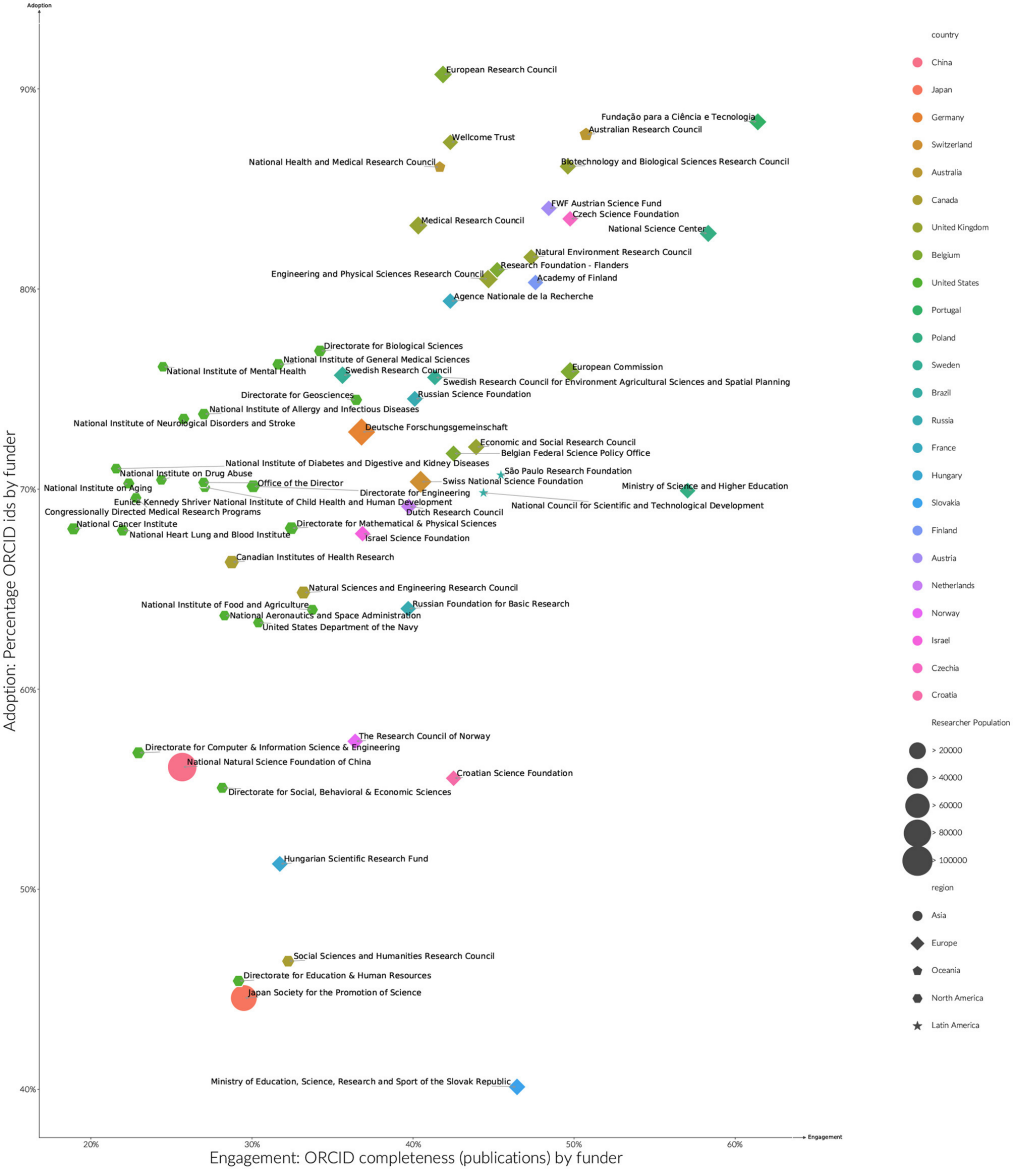
Estimated ORCID adoption and engagement by funder researchers > 5 years publication history, publications (2015–2019). Sizes indicate the number of researchers by funder, with shapes denoting world regions. To aid readibility of funder names, axis do not start at zero.

Figure 5 shows the top 60 funders by the number of researchers with ORCID iDs that they have funded between 2015 and 2019. Across these 60 funders, a much higher ORCID adoption rate can be observed for funded researchers than compared with country averages. This is to be expected to some degree, as there will be a greater overlap between researchers that receive funding, and researchers required to have an ORCID iD as part of publisher ORCID policies. A similar shift is not observed in the engagement rates by funder when compared to overall country rates.

Even with the overall increase in ORCID adoption rates, distinct funder patterns can be observed. The United Kingdom, Finland, Portugal, Australia, Austria and Czechia have very high adoption rates (between 80% and 90%). Many of these funders are associated with funder ORCID policies that either mandate, or strongly recommend the use of ORCID iDs in funder submissions. That engagement rates for these funders do not differ significantly from country norms, suggests an impact beyond just those who were funded to applicants and the broader community. An underlying information systems capacity for a country to accept a funder mandate may also be in play, with the United Kingdom, Australia, Finland and Portugal, and Czechia all having strong research reporting practices at the country and institutional level. High levels of research engagement implies a high level of ORCID record maintenance. Countries with a mature network of Institutional Current Research Information Systems will be better supported with these maintenance activities.

A separate band of funders including funders from the United States, Canada, Germany, Russia and Israel sees adoption rates between 60 and 80%. Within this second band, where identifiable in funder policies listed by ORCID (ORCID, 2020), ORICD integration funder appears to be more technical and optional rather than policy driven. Other funders within this band have more recently launched ORCID initiatives, the effects of which would not be seen in the analyzed period.

### 3.2. ORCID Adoption by Research Category

**Figure 6:**
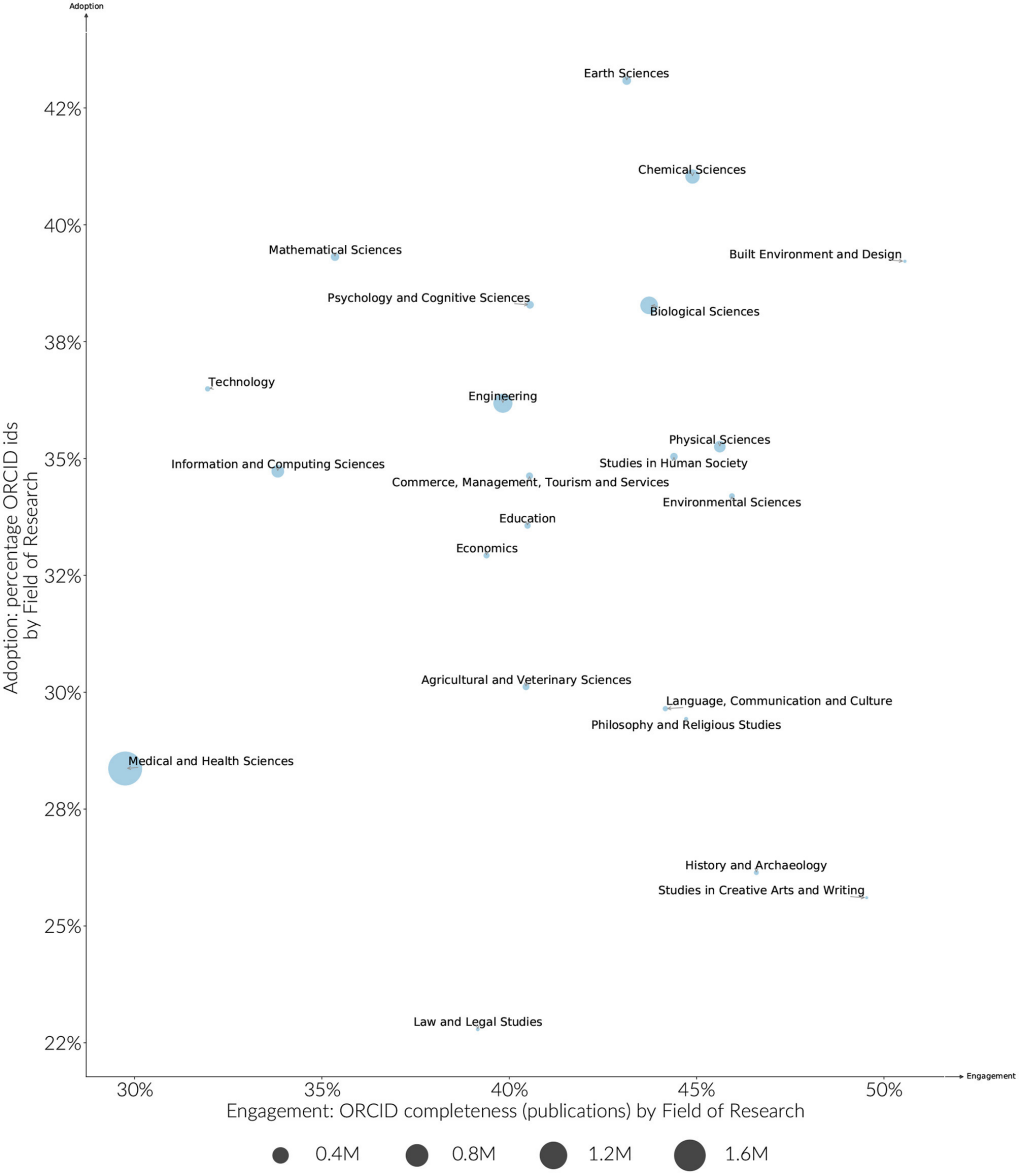
Estimated ORCID adoption and engagement by Field of Research researchers > 5 years publication history, publications (2015–2019). The size of the circle represents the size of the identified research population.

**Figure 7:**
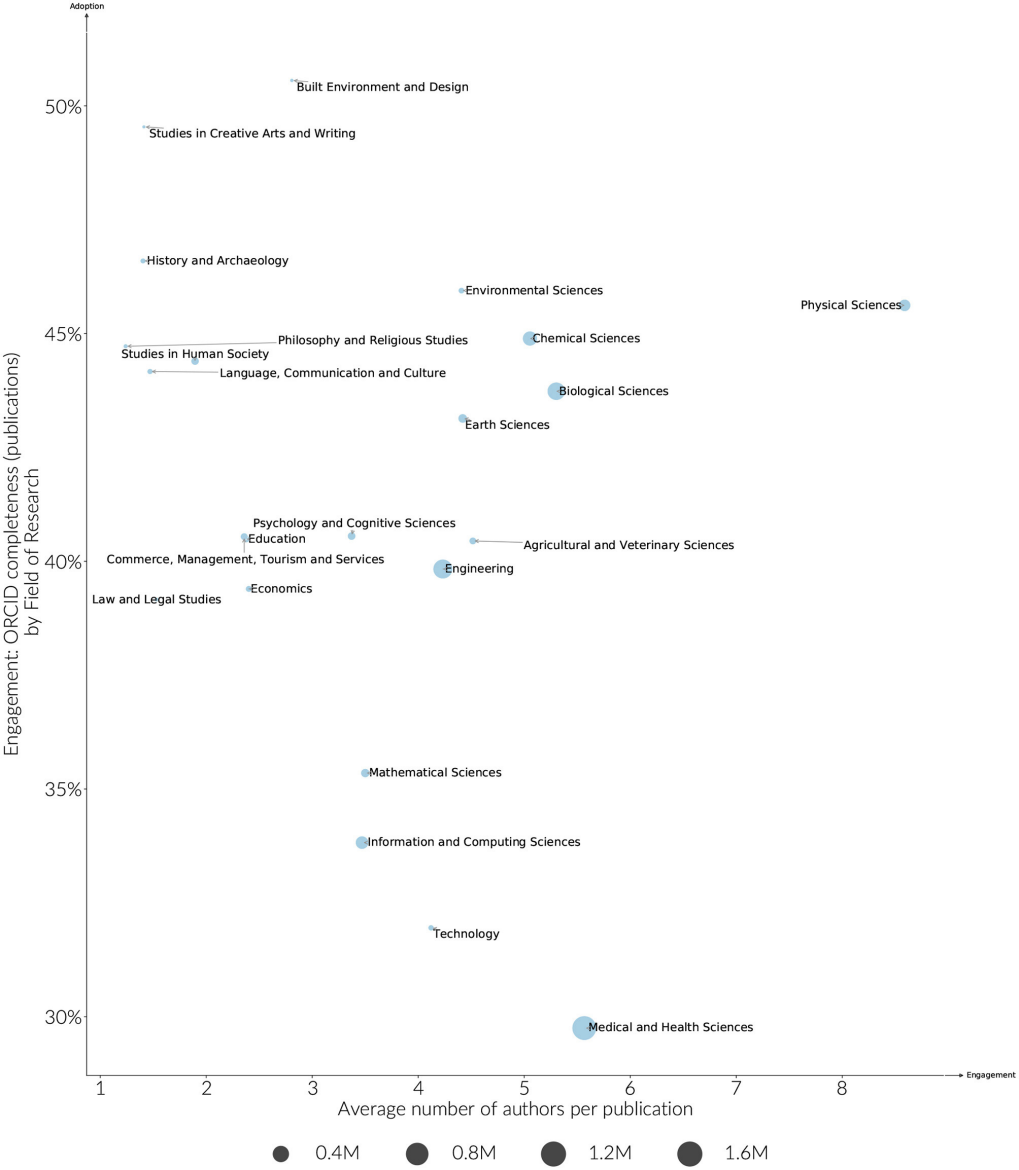
Estimated engagement by Field of Research compared to the average number of authors per paper. Researchers are included if they have > 5 years publication history, publications (2015–2019). The size of the marker indicates the size of the identified researcher cohort.

Overall, funder adoption and engagement rates are clustered more by country than they are by discipline, however some discipline effects can still be observed. Medically focused funders in particular have lower engagement rates on average when compared to other funders in the same country. These differences in discipline are also borne out more generally. As shown in Figure 6, ORCID adoption by discipline ranges 25–45%, and engagement from 30 to 50%. Earth Sciences and Chemical Sciences have both high adoption and engagement rates. Humanities research areas are distinguished by having lower adoption levels, but higher engagement levels. The large difference between adoption and engagement levels for these fields is partly explained by the articles in these fields having fewer authors per paper, and therefore fewer middle authors that are unlikely to receive ORCIDs given current publishing workflows. The average number of authors per paper does not explain the disparity in engagement across all disciplines, however. For instance, researchers in Medical and Health Sciences have a much lower engagement rate when compared to the relatively high adoption and engagement rates of disciplines with a similar average number of authors per paper such as Chemical or Biological Sciences (Figure 7).

As disciplines cross different country and funder environments, a high engagement and adoption level by discipline suggests that there are pockets of research practice that are closer to normalizing the use of ORCID for all authors.

### 3.3. ORCID Adoption - Publisher Level

Like funders, publishers support ORCID adoption and engagement via different mechanisms. ORCID adoption can be driven by publisher mandates. Engagement is supported most fully by providing all authors on a paper the opportunity to assert their ORCID iD. Publishers complete their responsibilities as research information citizens by passing the ORCID metadata through to Crossref.

**Figure 8:**
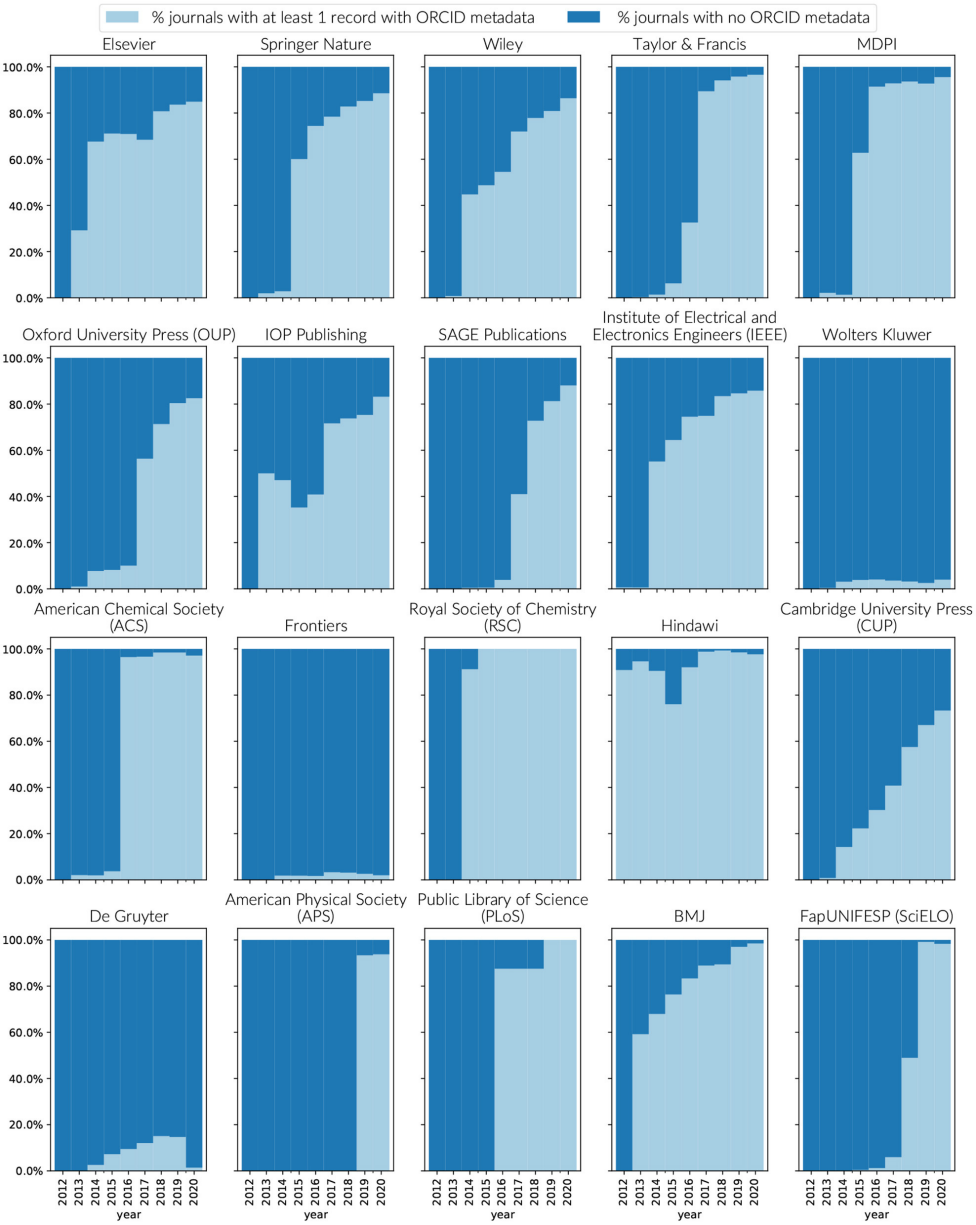
Journals supporting the use of ORCID's within Crossref metadata by publisher by year. To be counted as supporting ORCID, a journal must have at least one record in Crossref that includes an ORCID iD in its metadata. Source: Crossref public file matched to Dimensions.

With a few notable exceptions, support for ORCID in publication metadata by journals and publishers has increased significantly, particularly since 2016. For the top 16 publishers by volume, Figure 8 outlines the number of journals per publisher that have evidence of ORCID metatdata support in their Crossref records. With the exception of Wolters Kluwer, De Gruyter, and Frontiers, near complete journal support for expressing at least a minimum amount of ORCID metadata has either been reached, or there is a clear trend toward it. Presence of ORCID metadata in the Crossref records is not only a measure of publisher support adoption of ORCID, it is also a measure of community participation in open metadata that can be further consumed by downstream systems—a commitment outlined in the ORCID Open letter for publishers (ORCID, 2016). In the case of Frontiers, collection of ORCID iDs is a part of their workflow processes, however there was an oversight in passing the information across to Crossref [Internal Communication].

The level of support for ORCID iDs within publications by publisher is less uniform. In 2016, many publishers signed up to the commitment to require at least the corresponding author to connect their ORCID iD, with the understanding that all authors should be provided the option to assert their relationship to the paper (ORCID, 2016). Most publishers began their implementations by implementing the first requirement with support for additional authors proceeding at different paces (Meadows and Haak, 2017).

**Figure 9:**
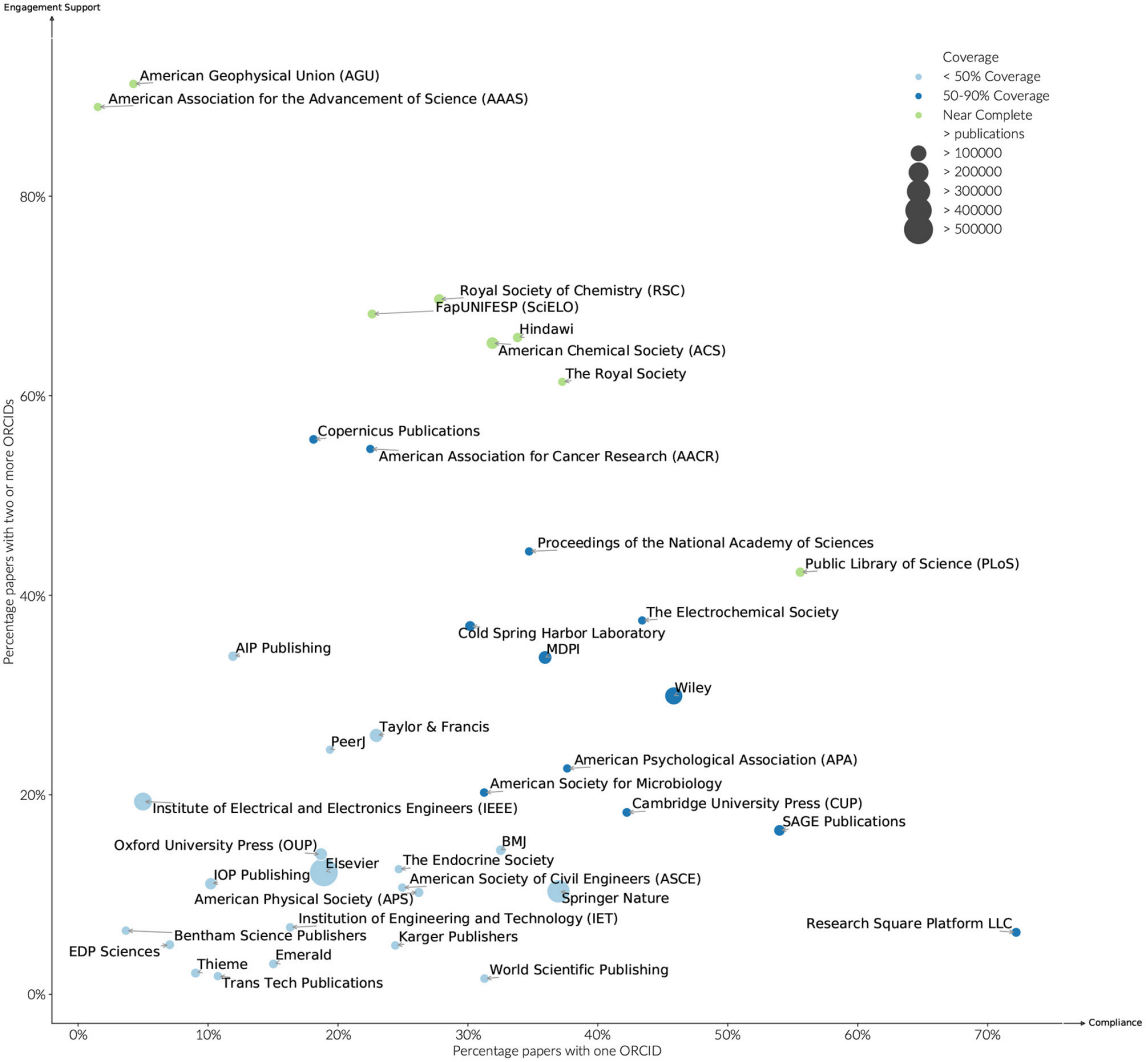
ORCID adoption and engagement by publisher, publications (2019).

By looking at papers published in 2019 with more than three authors, it is possible to observe how this trend has since moved. Examining the top 20 publishers by volume of ORCID assertions in 2019 (see Figure 9), the dominant publishing mode was still one ORCID iD per paper, however, clear differences in publishing practice can be observed. Nine publishers had at least one ORCID on over 90% of their publications in 2019. Of these JMIR, stands out both in the fact that it has the highest percentage of papers with two or more ORCID iDs, and that its overall discipline that it serves (Medicine and Health Services) does not have a high researcher engagement rate. Eight publishers had a percentage of greater than 60% papers with two or more ORCID iDs per paper, with a further band of seven between 20 and 40%. Elsevier and Springer Nature, the largest of the publishers have approximately 10% of their papers with two or more ORCID iDs, although their coverage of papers with one ORCID iD differs significantly at 18 and 38%, respectively. That there is such a difference in the spread of support for more than one ORCID suggests that the constraint still lies within individual publishing platform implementations, rather than a willingness for researchers to change behavior.

## 4. Discussion

In the previous section, a scientometric analysis of ORCID behavior reveals a research information citizenry that is serious about their obligations to each other, albeit one still in transition to ORCID-centric workflows.

We have shown that:

- In contrast to the internationalization of research, ORCID adoption and engagement patterns are regional, with countries such as Portugal, Poland, Denmark, and Australia leading the way and research giants such as the United States, China and Japan falling behind. Researchers within countries with low ORCID adoption rates are also more likely to be disengaged with their profile.
- ORCID adoption rates for funded researchers are significantly higher than their country averages, reflecting the influence of both publisher and funder mandates
- Publisher mandates have played a key role in encouraging ORCID adoption, however the capacity for researchers to supply ORCID iDs is now significantly outstripping publisher ability to record them as part of the submission process
- Publishers are meeting their responsibilities for distributed metadata stewardship around ORCID, however there remain some challenges in retrofitting new ORCID processes to existing submission workflows. These challenges resulted in an error rate of ORCID to author assertions of about .5% in 2020. Continued data quality monitoring is essential to ensure that this error rate continues to fall.
- ORCID adoption and engagement profiles differ significantly by research discipline, with Chemical Sciences and Earth Sciences having the highest rates, and Medical and Health Sciences the lowest. Moving beyond mandates, innovation in ORCID engagement by discipline provides a sustainable path for ORCID adoption going forward.

### 4.1. Addressing Researcher Disengagement

Critically, as might be expected, ORCID's success looks different by region, funding regime, and subject area. Each of these factors plays intimately with the likelihood of success of ORCID for a given researcher. If the researcher works in an established research economy in a high-income country with a dual-funding structure and national evaluation in a STEM research area then they are most likely to have both drivers to use ORCID and the opportunity to benefit from infrastructure investments. All this to say that depending on where in the world a researcher is based, they will likely have a significant difference on how integral is ORCID to their daily workflows.

For ORCID, Research Information Citizenship is not just about having an ORCID iD, but using it in expected ways. For a researcher, a key responsibility is not only ensuring that their information is kept up to date, it is also about ensuring that information can flow into their ORCID record with as little latency as possible. That countries with low engagement and adoption rates also exhibit a higher rate of disconnection between Crossref and ORCID is of significant concern. As publisher support for ORCID increases, these researchers are likely to experience the administrative burden of ORCID (which typically impact article submission workflows), without benefiting from the administrative benefits (which typically accrue during national evaluation or funding applications). Strategic engagement of these researchers will not only increase the local benefits of ORCID to the researchers involved, it also offers a path toward reducing the number of 'empty' ORCID profiles.

### 4.2. Emerging Strains Within Distributed Metadata Stewardship

On the other side of the relationship, it is remarkable that most publishers still publish more publications with only a single ORCID rather than multiple ORCIDs. Pressure to support ORCID assertions for all authors on a publication is mounting, with the capacity for researchers to supply their ORCID at the time of submission now outstripping functionality to support it.

Some journals are now choosing to implement ORCID policies that are beyond the current capacity of their publishing workflows Willighagen et al. (2019). For these journals, ORCID iDs will be supplied as part of the submission, however they will be unauthenticated by the researchers themselves, leaving open the possibility that a researcher could be misidentified. It is possible that initiatives designed to increase ORCID engagement could also break community trust by introducing errors into the system.

This pressure on publishers will increase still further with an evolution of funder requirements around open access publishing. UKRI now require all authors to be uniquely identified by their ORCID iD on papers published after April 2022 (UKRI, 2021). Notably, the policy does not specifically require ORCID iDs to be authenticated, raising the risk that the number of unauthenticated ORCIDS will rise significantly. This level of funder activism is interesting in that it imposes a mandate on coauthors from other countries to add their authenticated ORCID to UKRI funded publications.

Overall, publishers can be seen to be meeting their research information citizenship obligations by passing on metadata through to Crossref. The problem of author shuffling as identified in Section 2 reflects a persistent inherent difficulty with these workflows. At the core of the issue is the task of assigning ORCIDs to the individual author statements made through the manuscript submission process. Workflows that begin with the free text author statement on a manuscript and require a decision to be made on which author belongs to which ORCID. These decisions introduce name matching errors that are difficult to completely overcome, particularly when retrofitting ORCID to fit over legacy submission workflows. Continued monitoring of author shuffling with feedback to publishers to correct them should be considered an important activity to continue to maintain trust in the ORCID ecosystem.

### 4.3. The Importance of the Crossref Dataset When Measuring ORCID Adoption

When assessing the success of ORCID adoption and usage, we believe that we have demonstrated that it is not enough to assess the completeness of the ORCID registry in isolation. There have been many studies that compare the completeness and reach to research profiles such as ResearchGate (for example Boudry and Durand-Barthez, 2020). Because there are so many ORCID assertions in Crossref that have not made it back to the ORCID registry, this approach will almost certainly underestimate researcher ORCID engagement. By comparing ORCID to other profiling systems, such studies also risk incorrectly characterizing challenges with ORCID adoption as a choice between profile systems. This perspective leaves the research information citizenship that publishers and other actors exhibit in establishing the ORCID research graph unexamined.

### 4.4. Reflections on the Role of Scientometric Monitoring of ORCID Practices Going Forward

Scientometric monitoring of ORCID adoption and usage can offer insight into how ORCID practice is taking place in the community. Although providing an imperfect lens, by extending the known research graph through the use of natural language processing and algorithmic approaches, tools like Dimensions provide a way to observe these shifting dynamics, as well as make decisions about which interventions are likely to have the most impact in moving the research community forward. As illustrated by the ORCID journey, establishing new research practices centered around persistent identifiers require interconnected efforts to build new research infrastructure and change research practices. At different points of the journey, different approaches become possible. A first round of technical implementations for publishers focusing on connecting the first or corresponding author to their ORCID is now under pressure to accommodate all authors on a paper. What began as a push from publishers to make researchers supply their ORCID iDs is now reversing (in some disciplines) to be an expectation that all authors on a paper should be able to supply their ORCID. Through scientometric monitoring, we are able to identify these changes as they occur.

Scientometric monitoring can also play a role in selecting the most effective areas of research in which to innovate. Publisher support and innovation around ORCID may only just be beginning. Within disciplines where ORCID adoption and engagement levels are already high, it might also be possible to turn the relationship between author and ORCID on its head by adopting ORCID first approach to author assertions. Beginning with an ordered set of ORCID iDs, it would then be possible to derive the authorship statements on a paper. ORCID iDs could then be authenticated as part of the submission process (or as part of the document authoring process) without the additional requirement for author statement matching. Uncoupling ORCID author assertions from the submission process would also open up opportunities for greater collaboration between publishers and research authoring tools. Based on the observations made in this paper, it is more likely that innovations such as this would be more likely to take hold in fields with high ORCID adoption and completeness levels such as Earth Sciences or Chemical Sciences.

Of course, to be useful, the insights provided by scientometric analysis must be also by sufficiently accurate. Although aspects of this study, particularly the completeness calculations, would benefit from replication using data sources other than Dimensions, a level of calibration can be observed in the results themselves. For instance, the impact of ORCID interventions at the country level can be clearly recognized in the analysis above.

Finally, whilst this analysis has only measured funder contributions to ORCID adoption and engagement rates indirectly, funder interventions can be seen to correlate with high ORCID and engagement rates - particularly amongst countries with well established networks of current research information systems. Until recently, funders have expressed their role as a researcher information citizen as a consumer of ORCID information. More recently (ORCID Funder Working Group, 2019), in 2019 a move analogous to the publisher open letter (ORCID, 2016) a consortia of funders has proposed extending their role to also be a creator of ORCID assertions for grants, by creating both a public record of the grant with a DOI, and an ORCID assertion to it. As these new information pathways establish, and the known research graph continues to expand (Cousijn et al., 2021), scientometric approaches such as the one showcased here will provide an important methodology for charting its progress.

## Data Availability Statement

The datasets presented in this study can be found in online repositories. The names of the repository/repositories and accession number(s) can be found below: doi: 10.6084/m9.figshare.16638337.

## Author Contributions

All the work contained in this article was conceptualized, carried out, and written by SP.

## Conflict of Interest

SP was employed by Digital Science.

## Publisher's Note

All claims expressed in this article are solely those of the authors and do not necessarily represent those of their affiliated organizations, or those of the publisher, the editors and the reviewers. Any product that may be evaluated in this article, or claim that may be made by its manufacturer, is not guaranteed or endorsed by the publisher.

## Footnotes

^1^The idea of a centralized identity and authentication mechanism for academia is an alluring one. However, the idea that, at the current time, publishers, funders and academic institutions would all make themselves reliant on a centralized third-party is difficult to imagine. This is fundamentally counter-cultural in an academic context. Furthermore, we live in an era where the direction of movement in technology is toward the decentralization of trust or, more specifically, the distribution of trust across networks. Hence, it seems unlikely that centralization in this context would be a wise structural choice at this time.
